# Molecular Research and Treatment of Breast Cancer: From EMT Regulation to Innovative Therapeutics

**DOI:** 10.3390/ijms27020659

**Published:** 2026-01-09

**Authors:** Anna Kawiak

**Affiliations:** Intercollegiate Faculty of Biotechnology, University of Gdansk, Abrahama 58, 80-307 Gdansk, Poland; anna.kawiak@biotech.ug.edu.pl

Breast cancer is the leading cause of cancer-related deaths in women [1]. The heterogeneity of the disease determines its progression, prognosis, and response to treatment. It is also a major factor underlying therapeutic resistance and recurrence [2]. Recent molecular research featured in this Special Issue has provided important insight into pathways facilitating this heterogeneity, including epithelial–mesenchymal transition (EMT), tumor-microenvironment interactions, metabolic adaptations, and cell-cycle checkpoint control. Collectively, this research highlights therapeutic strategies targeting EMT reversal, microenvironment modulation, metabolic vulnerabilities, and checkpoint inhibitors as potential means to enhance clinical outcomes in breast cancer.

Epithelial-to-mesenchymal transition (EMT) is a key process contributing to breast cancer progression during which cells lose epithelial characteristics associated with E-cadherin expression, leading to loss of cell–cell adhesion, changes in cell polarity, and cytoskeletal reorganization. Cells acquire mesenchymal properties, including increased motility, invasiveness, and therapeutic resistance [3]. Recent studies demonstrate that cancer cells do not necessarily exist in discrete epithelial (E) or fully mesenchymal (M) states but instead can inhabit a stable intermediate phenotype termed the mixed or hybrid epithelial–mesenchymal (E/M) state. The hybrid E/M state is particularly significant because cancer cells within it exhibit greater tumorigenicity, including enhanced tumor initiation and growth. The hybrid E/M phenotype is regulated by various transcription factors, among which SNAIL and ZEB1 are often crucial determinants of the various EMT states, with hybrid cells frequently co-expressing both (distinguishing them from epithelial cells, typically negative for ZEB1, and mesenchymal cells with high ZEB1 but lower SNAIL) [4]. The reciprocal feedback loops between “miRNA-200c-3p-ZEB1” and “miRNA-34a-5p-SNAIL” constitute core EMT networks regulating this phenotypic plasticity [5]. Recent findings by Merckens et al. from breast epithelial–cancer hybrids demonstrate that ZEB1 knock-out in these cells reveals a more complex regulatory network than previously understood: ZEB1 depletion did not correlate with downregulation of EMT-related markers like N-CADHERIN and VIMENTIN, or with substantial upregulation of miR-200c-3p. However, the study observed a significant reduction in colony and mammosphere formation, alongside a marked increase in ALDH1-positive cells (a mammary stem cell marker) in certain hybrid-KO variants. These results indicate that ZEB1-associated cancer stem cell (CSC) characteristics and EMT-related properties involve redundant mechanisms. Higher SNAIL and ZEB2 expression levels in ZEB1 knock-out (KO) cells suggest that these factors might compensate for ZEB1’s loss, thereby maintaining mesenchymal traits and partial stemness. The moderate, cell-line-specific phenotypic changes post-ZEB1 knock-out point out the robustness of the hybrid E/M state in tumor hybrids, potentially driven by cell-fusion-induced mechanisms. These findings highlight the therapeutic challenges in targeting single EMT transcription factors for metastasis prevention, as the system exhibits compensatory pathways and phenotypic plasticity [Contribution 1].

In addition to cell-intrinsic regulatory programs, exosome-mediated communication is a critical extrinsic driver of breast cancer heterogeneity. Exosomes are crucial extracellular vesicles that significantly influence breast cancer (BC) progression, metastasis, and drug resistance by mediating intercellular communication and transferring various biological components, including lipids, proteins, and RNAs. These vesicles play a pivotal role in tumor development by promoting immune suppression, cell proliferation, angiogenesis, and tumor cell invasion and migration. A critical mechanism through which exosomes contribute to tumor progression is the promotion of EMT. For instance, exosomal miR-18b from cancer-associated fibroblasts (CAFs) stimulates NF-κB activation and promotes nuclear Snail ectopic activation, which induces EMT, thereby driving cellular metastasis and invasion. Breast cancer itself exhibits high heterogeneity, classified into several subtypes based on genetic and clinical characteristics. This heterogeneity is further influenced by exosomes, which contain rich biological information and reflect the cell-of-origin phenotype. Specific miRNA expression profiles in exosomes can differentiate BC subtypes, including HER2-positive and triple-negative breast cancer (TNBC). Furthermore, exosomes are involved in drug resistance, facilitating drug efflux and inactivation, and contributing to chemoresistance and hormone resistance. Given their rich biological information and involvement in critical processes like EMT and the modulation of tumor heterogeneity, exosomes are promising as diagnostic and prognostic biomarkers and as tools for targeted drug delivery in personalized therapy [Contribution 2].

Recent research further highlights the therapeutic relevance of targeting exosome-mediated pathways by identifying peptide-based strategies that directly disrupt vesicle biogenesis and EMT-associated signaling. The study by Huang et al. specifically investigated the HIV-1 Nef-derived Secretion Modification Region (SMR) peptide, demonstrating its direct interaction with the chaperone Mortalin (HSPA9) and the intermediate filament protein Vimentin, both of which are associated with EMT regulation, cytoskeletal dynamics, and tumor aggressiveness. Through surface plasmon resonance (SPR) and functional assays, the authors showed that SMR peptides and specific Mortalin-derived antagonistic peptides effectively interfere with Mortalin-Vimentin complex formation. This interference leads to a significant suppression of tumor-derived exosome release in breast cancer cell lines. Importantly, these interactions are shown to shift vesicle cargo and trafficking away from EMT-promoting programs, advancing a partial reversion towards a more epithelial state. By functionally linking EMT regulation, cytoskeletal remodeling, and exosome secretion via the Mortalin–Vimentin-associated network, this study proposes the use of short linear peptide motifs to disrupt communication networks that sustain breast cancer heterogeneity, metastatic competence, and therapy resistance [Contribution 3].

In addition to EMT and microenvironmental regulation, metabolic reprogramming offers another therapeutic avenue. Glutamine metabolism, a key metabolic process supporting the bioenergetic and biosynthetic demands of cancer cells, is emerging as a potential therapeutic target [6]. Using the amino acid analogs Acivicin and Azaserine, Abdelsattar et al. demonstrated selective inhibition of glutamine synthetase (GS) activity in MCF-7 breast cancer cells. Glutamine depletion was associated with suppression of cell proliferation and induction of apoptotic signaling, without detectable toxic effects on non-tumorigenic breast epithelial cells. Mechanistically, GS inhibition was associated with reduced Raf-1 signaling, restoration of tumor suppressors PTEN and p53, and a shift toward an anti-inflammatory cytokine profile and reduced TNF-α production. Notably, both compounds exhibited strong binding affinity for GS, supporting direct enzymatic targeting as the basis for their anticancer activity and suggesting that targeting metabolic enzymes may offer therapeutic strategies to disrupt metabolic dependencies in breast cancer cells [Contribution 4].

In parallel with metabolic targeting strategies, disruption of cell-cycle checkpoint control has emerged as a promising approach in the treatment of breast cancer. WEE1 kinase, a key regulator of the G2/M and intra-S phase checkpoints through inhibitory phosphorylation of CDK1 and CDK2, is increasingly recognized as a therapeutic target, particularly in tumors with high replication stress or defective G1/S checkpoint control. The review of Zhang et al. highlights that breast cancers, especially triple-negative and therapy-resistant subtypes, frequently show increased reliance on WEE1 activity to survive DNA damage and replication stress. Pharmacological inhibition of WEE1 induces premature mitotic entry, replication stress, and mitotic catastrophe, thereby sensitizing tumor cells to chemotherapy and PARP inhibitors and possibly increasing responses to immune-modulating treatments. Importantly, emerging data suggest that WEE1 inhibition may also target cancer stem-like populations and enhance antitumor immune responses through activation of the STING pathway. Although clinical translation has been challenged by toxicity and variable patient responses, the development of next-generation WEE1 inhibitors and biomarker-guided combination strategies demonstrates the importance of checkpoint targeting as a complementary approach to breast cancer treatment [Contribution 5].

The studies presented in this Special Issue collectively demonstrate that breast cancer heterogeneity is driven by diverse mechanisms. These include phenotypic plasticity, which enables cancer cells to adapt and change their characteristics, and exosome-mediated intercellular communication, which facilitates complex interactions within the tumor microenvironment. Additionally, metabolic reprogramming and cell-cycle dependencies play crucial roles in shaping tumor behavior and progression. Given the complexity and redundancy observed in breast cancer, the Special Issue stresses the critical role of developing multifaceted therapeutic approaches. The research and therapeutic strategies presented strongly support an integrated framework for combination therapies and biomarker-guided precision medicine to overcome resistance and improve outcomes in breast cancer treatment.
